# GreeningDB: A Database of Host–Pathogen Protein–Protein Interactions and Annotation Features of the Bacteria Causing Huanglongbing HLB Disease

**DOI:** 10.3390/ijms221910897

**Published:** 2021-10-08

**Authors:** Cristian D. Loaiza, Naveen Duhan, Rakesh Kaundal

**Affiliations:** 1 Department of Plants, Soils and Climate, Utah State University, Logan, UT 84322, USA; cdloaiza@aggiemail.usu.edu (C.D.L.); naveen.duhan@usu.edu (N.D.); 2 Bioinformatics Facility, Center for Integrated BioSystems, Utah State University, Logan, UT 84322, USA; 3 Department of Computer Science, Utah State University, Logan, UT 84322, USA

**Keywords:** citrus greening disease, huanglongbing, citrus, database, protein annotations, host–pathogen interactions, protein–protein interactions

## Abstract

The *Citrus* genus comprises some of the most important and commonly cultivated fruit plants. Within the last decade, citrus greening disease (also known as huanglongbing or HLB) has emerged as the biggest threat for the citrus industry. This disease does not have a cure yet and, thus, many efforts have been made to find a solution to this devastating condition. There are challenges in the generation of high-yield resistant cultivars, in part due to the limited and sparse knowledge about the mechanisms that are used by the *Liberibacter* bacteria to proliferate the infection in *Citrus* plants. Here, we present GreeningDB, a database implemented to provide the annotation of *Liberibacter* proteomes, as well as the host–pathogen comparactomics tool, a novel platform to compare the predicted interactomes of two HLB host–pathogen systems. GreeningDB is built to deliver a user-friendly interface, including network visualization and links to other resources. We hope that by providing these characteristics, GreeningDB can become a central resource to retrieve HLB-related protein annotations, and thus, aid the community that is pursuing the development of molecular-based strategies to mitigate this disease’s impact. The database is freely available at http://bioinfo.usu.edu/GreeningDB/ (accessed on 11 August 2021).

## 1. Introduction

Citrus greening disease (or huanglongbing) is the most devastating condition affecting citrus fields around the globe. Within the last few years, the citrus industry has been heavily affected due to the rapid spread of huanglongbing (HLB). In the US alone, the disease has caused a 21% decrease in the fresh citrus fruit market and around 71% in the production of oranges [1]. This infectious condition triggers when a Gram-negative bacterium *Candidatus* Liberibacter gets established into the phloem of a healthy citrus tree. *Ca*. Liberibacter bacteria are transmitted into citrus trees through the insect vector *Diaphorina citri* (also known as the Asian citrus psyllid), which carry the bacteria and release them into the trees during the feeding process. Infected trees typically have asymmetrical blotchy mottle, yellow shoots, and partially green, lopsided fruits [2]. Three species of *Liberibacter* genus have been identified as citrus pathogens, named according to the continent from where they were originally discovered: *Ca*. Liberibacter asiaticus, *Ca*. Liberibacter americanus*,* and *Ca*. Liberibacter africanus. Within this genus, there is another major pathogenic species (*Ca.* Liberibacter solanacearum) that although does not seem to be affecting citrus, is a causative agent of zebra chip disease [3]. Except for *L. crescens,* which is a non-pathogenic strain [4], no other *Ca*. Liberibacter strains have been successfully cultured in artificial media, making the possibility to apply traditional molecular and genetic analysis difficult.

Notwithstanding, research groups have been able to elucidate some of the characteristics behind HLB infection. Now, it is well known that *Ca*. Liberibacter is an obligate parasite that lacks some of the housekeeping genes of regular bacteria, meaning that this bacterium is only able to multiply if it is inside a eukaryotic host, either a plant or a psyllid [5]. Furthermore, some molecular mechanisms used by HLB bacteria to overcome plant immune systems have been elucidated, which includes increasing the levels of salicylic acid in plants [6], *Liberibacter* prophages assisting in the suppressing of *Citrus* plant defenses [7], and the induction of the expression of immune response genes, such as those from the mitogen-activated protein kinase signaling pathway [8]. These processes often involve the prompt of protein–protein interactions (PPIs). PPIs constitute an essential part of all biological processes within the cell for all living beings. PPIs can be classified as intraspecies, within an organism; or interspecies, involving at least two organisms. Interspecies interactions are critical to understanding the molecular basis of pathogenesis between host plants and their pathogens [9,10].

Identifying the citrus proteins targeted by HLB bacteria is fundamental to advance into better management of this disease. There are few resources available to retrieve PPI data for many plants in terms of host–pathogen systems [11,12]; unfortunately for citrus, except for the study on the citrus targets of an SDE1 effector from *Ca*. Liberibacter asiaticus [13]*,* there is no publicly accessible information of inter-species PPIs. Implementation of databases incorporating host–pathogen interaction features have been used to bolster knowledge about the disease, thus accelerating the development of novel drugs and therapeutics [14,15]. Furthermore, the development of genomic databases is crucial to the generation of advanced molecular approaches. For citrus genomics, there is a Citrus Genome Database (CGD) [16] available, a comprehensive resource with various modules that is helpful in enhancing studies on citrus breeding and genomics. Databases alike are also implemented in virulent bacteria [17,18]; however, no such tool exists for the *Liberibacter* genus.

To deliver a platform to facilitate the study of HLB bacteria, we have developed GreeningDB, a database implemented to store and offer the annotation of *Liberibacter* strains proteomes, and a novel host–pathogen ‘comparactomics’ tool that allows users to compare predicted host–pathogen interactomes of *Citrus* and HLB bacteria. GreeningDB is built to deliver a user-friendly interface, including network visualization and links to external annotation resources. This database can serve as a central resource for scientists, and the community in general, who are pursuing development of molecular-based strategies to mitigate the impact of citrus greening disease. GreeningDB is freely accessible through http://bioinfo.usu.edu/GreeningDB (accessed on 11 August 2021).

## 2. Results

### 2.1. GreeningDB Overview

The GreeningDB database comprises 10 citrus species and 25 *Ca*. Liberibacter strains. The database contains five tabs: Home, Tools, Features, Datasets, and Help. The “Home” page gives an overview of the database and the host–pathogen species that are present. The “Tools” page has three tools: interactome comparison, a sophisticated search module, and BLAST search. The “Features” tab connects to nine distinct features pages: host–pathogen interactions, protein annotations, secreted effectors, subcellular localization, gene ontology, functional domain mapping, Citrus genes altered by HLB infection, virulence effectors, and predicted effectors. The “Datasets” page contains genomic information for citrus species and strains of *Ca*. Liberibacter.

### 2.2. GreeningDB Tools

#### 2.2.1. Novel Comparactomics Tool

The main feature of *GreeningDB* is the comparison of host–pathogen interactomes. The comparison tool functions in two ways: the user may compare a citrus host to two *Liberibacter* strains, or they can compare two citrus hosts to one *Liberibacter* strain. The interolog method implemented in this comparison tool is designed in such a manner that it can anticipate whole-genome protein–protein interactions in a matter of seconds. It also allows users to tailor their search by selecting protein–protein interaction databases and a configurable BLAST alignment search criteria. When a user starts a comparison, a unique job ID is generated to allow the user to track the progress. Users can also receive an e-mail upon completion of the job by providing an e-mail address in the given box. When the task is completed, the results are shown in a tabular format, allowing the user to sort the table by column or filter the data using keywords. The network visualization page includes links to the NCBI database for chosen node proteins, as well as descriptions and degrees. Users may also use the force atlas functionality to alter the network layout and output network as an SVG image or JSON format. The search results for each unique job ID are kept on the server for 30 days.

#### 2.2.2. BLAST Search Tool

The standalone version of the NCBI BLAST is implemented on *GreeningDB* to provide the homology search functionality. All host–pathogen proteomes used to build *GreeningDB* are also available as BLAST databases with all *Citrus* susceptible to HLB, all *Citrus* tolerant to HLB, all *Ca*. Liberibacter infecting *Citrus,* or all *Ca*. Liberibacter infecting *Solanacearum*. A nucleotide or amino acid sequence may be uploaded by users and the system automatically determines the BLAST variant to use from BLASTp or BLASTx. The BLAST results page provides options to visualize alignments in a tabular or standard format. A more detailed version of the results is also implemented using BlasterJS [19].

#### 2.2.3. Advanced Search Module

*GreeningDB’s* advanced search module provides an interface via which users may search for information filtered by various keywords and other parameters, such as protein length, genomic range, and subcellular localization in a certain species, and download the results as a tab-delimited file. This search module is extensive, allowing searching for scholarly papers, protein annotations, GO keywords, experiment descriptions, and a variety of additional data that matches the searched keyword. Furthermore, a more basic option for doing a rapid search of a protein accession is accessible throughout GreeningDB, and both the “advanced” and “basic” searches will reveal all of the information that may be gathered from our database records.

### 2.3. Features in GreeningDB

Data acquired from the literature or results from the different annotation pipelines are given in various search modules; *GreeningDB* comprises a total of nine search modules. Several categories of *Liberibacter* protein annotations (protein annotations, secreted effectors, subcellular localization annotation, gene ontology (GO) term annotation, functional domain mappings (InterProScan), virulence factors, and predicted effectors (EffectiveDB)) can be retrieved from seven of those modules. In addition, two additional modules (citrus genes regulated by HLB infection, and host–pathogen interactions) are provided to collect annotations on citrus proteins discovered to be linked to HLB by literature mining. The module data will be shown in accordance with the dataset chosen. The dataset options within the *Liberibacter* annotation modules can also be set to specify which specific strain of *Liberibacter* (Table 1) to display, whereas in the other two modules, there is an option to specify from which host species to display annotation (*Citrus sinensis* or *Citrus clementina*) and the two host species from which the majority of the manual annotations were retrieved.

## 3. Discussion

**Figure 1:**
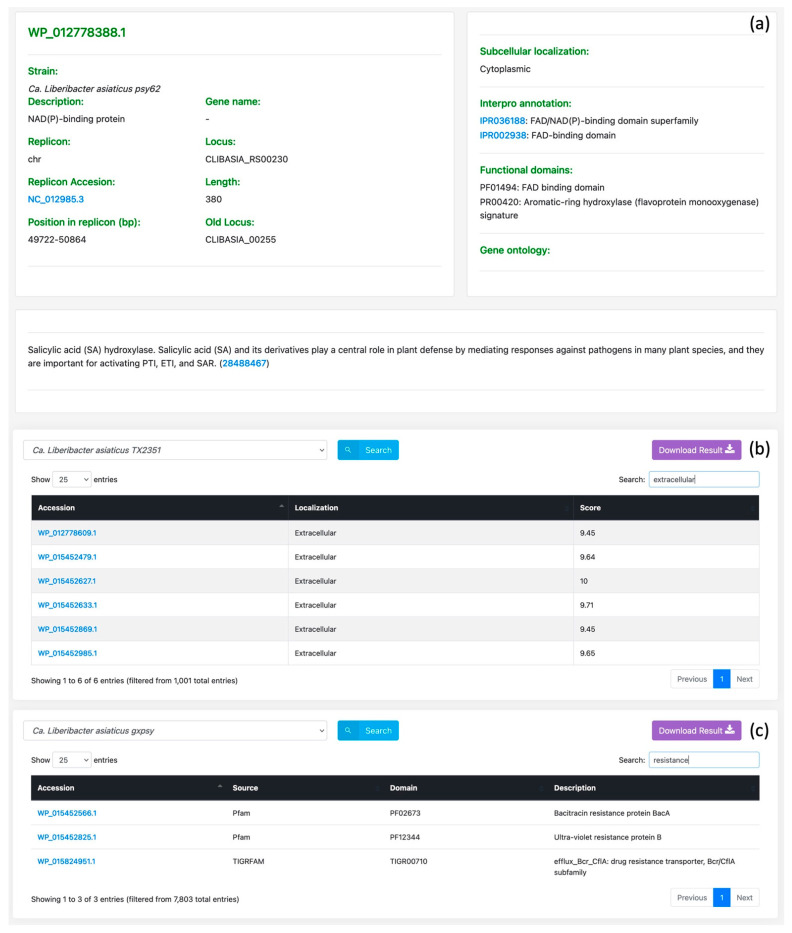
Examples of the annotation features of the GreeningDB search modules. (**a**) Description of WP_012778388.1, a ‘*Ca*. Liberibacter asiaticus’ str. psy62 protein; (**b**) Proteins that are located extracellularly in ‘*Ca*. Liberibacter asiaticus’ str. TX2351; (**c**) Proteins found with a “resistance” functional domain in ‘*Ca*. Liberibacter asiaticus’ str. gxpsy.

With a resource such as GreeningDB available, numerous activities may now be accomplished that were hard to complete previously. In the GreeningDB user-friendly interface, users can easily retrieve annotation of a *Ca*. Liberibacter asiaticus protein (Figure 1a); *C. sinensis* genes found regulated under HLB disease conditions (Figure 1b); secreted effectors of a *Ca*. Liberibacter africanus strain; obtaining proteins from a particular *Ca*. Liberibacter asiaticus strain that have “resistance” functional domains (Figure 1c); and extracellular proteins of a certain *Ca*. Liberibacter asiaticus strain, etc. By offering these functionalities inside GreeningDB, this database can become a primary resource for retrieving (and submitting) HLB-related protein annotations.

### 3.1. Host–Pathogen Interactome Comparison: In the Direction of a Better Knowledge of HLB Infection Mechanisms

Computational approaches for predicting protein–protein interactions have grown in popularity in recent years because conventional methods such as yeast two-hybrid [36] and co-immunoprecipitation [37] are time-consuming and expensive when used on a large scale. Furthermore, the use of computational techniques to predict PPIs has been shown to occasionally be more accurate than traditional strategies [38]. It is critical to account for variations in host–pathogen interactomes because strain-specific PPI patterns may play a critical role in the creation of strain-optimized treatments.

Similarly, there are variations in HLB development among citrus species [39], distinctions that may be significant and worth identifying because they may point to genes that should be targeted from an HLB-tolerant variety to increase resistance in a susceptible one. Our innovative comparactomics module has been deployed to assist researchers in identifying PPI patterns that may be unique or common among the various HLB systems. To the best of our knowledge, this is the first report of a tool capable of performing this HPI comparison study, which we refer to as *comparactomics*.

**Figure 2:**
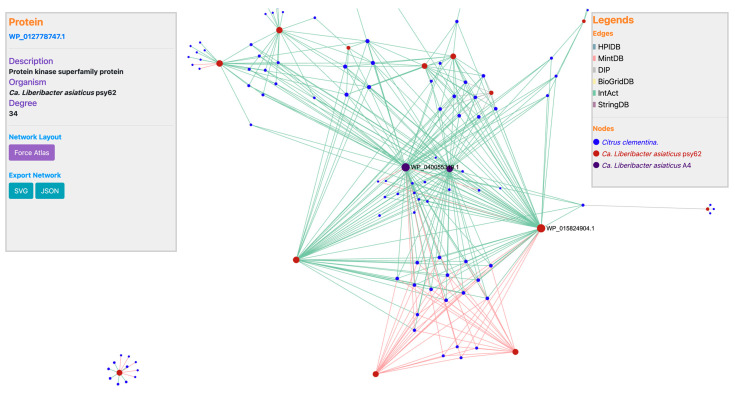
Host–pathogen interaction network visualization obtained through GreeningDB’s *comparactomics* module. *Citrus clementina* vs. ‘*Ca*. Liberibacter asiaticus’ str. psy62 interactome is compared against *Citrus clementina* vs. ‘*Ca*. Liberibacter asiaticus’ str. A4 interactome.

This novel *comparactomics* tool offers two types of comparisons. A user can compare two hosts (*Citrus*) to a pathogen (*Liberibacter*) or two pathogens (*Liberibacter*) to a single host (*Citrus*). This tool supports the host and pathogen datasets specified in the “Data collection” section. In this tool, the user may also choose which protein–protein interaction database to utilize as a template in the prediction process, as well as configure BLASTp alignment filters to find homolog proteins. GreeningDB also includes a network visualization platform built using SigmaJS; this plugin was specifically chosen for its efficiency in showing huge networks. A user may visualize a collection of attributes for each node (species, description, degree) from the host–pathogen network visualization of a typical comparison result; moreover, a user can quickly locate hub nodes (nodes with a higher number of edges) as shown in Figure 2.

This is beneficial because hub nodes have been discovered to be critical in understanding several infectious disease pathways [40]. The network analysis offered by our database is not restricted to the user; the resulting network files may be downloaded and viewed in any third-party network analyzer program that can handle JSON or tabular network files.

In biological networks, it is typical to identify proteins that interact with several proteins at the same time, and knowing their role can help to obtain a better understanding of the pathogenicity processes employed by the *Liberibacter* bacteria to resist citrus defenses. The variability in HPI patterns among a set of *Liberibacter* strains can cause differences in virulence and infection processes; thus, we strongly believe that providing proteomes from commonly occurring strains, as well as strains that do not appear to infect Citrus (e.g., *L. crescens* and *Ca*. Liberibacter solanacearum), will improve our understanding of this disease.

### 3.2. GreeningDB’s Future Development and Limitations

Because of existing limitations in genome assembly and annotation availability, some characteristics are not available to a few species in the database. Because studies on HLB molecular processes are often conducted using more refined genome assemblies, such as *C. clementina* and *C. sinensis*, there is little information available on Citrus genes regulated by HLB in other species. In terms of the pool of genomes available in *GreeningDB*, we expect to add more *L. crescens* strains, proteomes, and resistant Citrus datasets in the future, in addition to *P. trifoliata* and *C. excavata*. We were additionally constrained because we did not wish to include capabilities that were already available in other citrus genome resources, for example, the visualization of genomic variants or high-throughput sequencing experiments via JBrowse or citrus metabolic pathways using BioCyC. We will most likely collaborate with these citrus databases in the future or update the present version of those genomic tools in our database.

One of the most difficult challenges for a community-run database such as *GreeningDB* is its long-term viability, particularly in terms of content updates. To ensure that *GreeningDB* remains relevant to the community, we will update our database once a year, which includes upgrading the backend proteome files (both host and pathogen species) and then updating the backend HPI databases with the latest version (e.g., new version of HPIDB, STRINGdb, MINT, BioGRID, and other databases). Similarly, we will undertake an annual curation of HLB publication material in order to incorporate that data into our database records, as well as implement new features, such as HLB-related QTL and genetic markers in the second version of *GreeningDB*.

Depending on the influx of users and their specific requirements, the next version of the *GreeningDB* database might also include an API to access the database contents programmatically.

## 4. Materials and Methods

### 4.1. Data Collection

*Citrus* spp. and *Liberibacter* spp. proteomes were acquired from various sources. The *Citrus sinensis* Annotation Project (CAP) [41] was used to download the proteomes of *Fortunella hindsii* [42], *Citrus reticulata*, *Citrus grandis*, *Citrus ichangensis, Citrus medica,* and *Atalantia buxifolia* [43]. Protein sequences of *Citrus clementina* and *Citrus sinensis* [44] were obtained from Phytozome [45].

Citrus ecotypes exhibiting resistance to HLB infection include *Poncirus trifoliata* and *Clausena excavata* [46]; however, proteomes for these species were not available at the time this database was created. Nonetheless, we added protein sequences from both species to create a more comprehensive *GreeningDB*. TransDecoder [47] was used to transform the transcriptome of *Clausena excavata* [48] and the unigenes of *Poncirus trifoliata* [16] into protein sequences.

The proteomes of *Ca*. Liberibacter were acquired from the NCBI (https://www.ncbi.nlm.nih.gov/, accessed on 11 November 2018) (Table 1). This collection contains 13 *Ca*. Liberibacter asiaticus strains, 1 *Ca*. Liberibacter africanus strain, and 2 *Ca*. Liberibacter americanus strains. As a negative control, 8 *Ca*. Liberibacter solanacearum strains, as well as the *L. crescens* BT-1 proteome, are included in this resource. The proteome of the insect vector *Diaphorina citri* was also incorporated into *GreeningDB*, and psyllid protein sequences (*D. citri* OGS v2.0) were obtained from the Citrus Greening Solutions database [49].

### 4.2. Protein–Protein Interaction Database Collection

We utilized several PPI databases from The International Molecular Exchange Consortium (IMEx) [50] as templates to construct the interolog method in the comparactomics module. In the host–pathogen comparactomics module, *GreeningDB* uses the five major IMEX datasets: HPIDB v3.0 (2019) [12], an inter-species interaction database including four intra-species interaction datasets, IntAct (20 November 2018) [51], DIP (November 20, 2018) [52], MINT (20 November 2018) [53], and BioGRID v.3.5.169 [54]. STRINGdb [55] was also added to improve sensitivity in wide-range tests.

### 4.3. Comparactomics: A Host–Pathogen Interactome Comparison Tool

We developed a new module called the *comparactomics* tool to compare interactions between two distinct sets of HPIs. An interolog method was used within *GreeningDB* to achieve this on the backend. The interolog technique is based on the idea of transferring protein–protein interactions across comparable systems. For example, if A and A’ are orthologs and B and B’ are orthologs, then the interactions between A and B (in one system) and A’ and B’ (in another system) are interologs [56]. The ortholog proteins produced because of this approach are then used as a query to search the PPI databases. If a match in the PPI database matches to a host and pathogen protein, that protein pair is expected to interact. *GreeningDB’s* backend uses this approach to anticipate host–pathogen PPIs between *Citrus* and *Liberibacter* proteins. In fact, each proteome was matched to each of the six PPI databases independently, yielding six alignment result files per proteome. BLASTp was run using the default settings to align the sequences. Following alignment, further steps for interolog prediction are performed using our in-house R scripts that call the SQL functions; all of this occurs when a user submits a task. SQL tables representing PPI databases were indexed for interactor A and interactor B columns to speed up the ortholog match searching process.

### 4.4. Pathogen Protein Annotation

The full protein descriptions of *Liberibacter* proteins were obtained from the NCBI Genome Assembly and Annotation Report’s “Protein Details.” Conserved domain areas and Gene Ontology (GO) terms were predicted using InterProScan [57] with the “iprscan” option in all bacteria proteomes for functional annotation. Using PSORTdb [58], we were also able to determine the subcellular localization of all of the bacterium proteins. In addition, we ran EffectiveDB [59] to predict effectors for all *Liberibacter* strains.

### 4.5. Secreted Effectors and HLB-Related Proteins from Literature

To refine the annotation data received from the online sites and tools, we examined the literature for information on proteins or genes associated with citrus greening disease. Much of the data gathered comes from a limited number of articles in particular. For example, in a thorough research to predict *Liberibacter* Sec-dependent extra cytoplasmic proteins [60], the authors predicted signal peptides from diverse *Liberibacter* strains by integrating the findings of four signal peptide prediction methods. Another paper that used the data employed a bioinformatics process to predict 28 potential effector proteins from the *Ca*. Liberibacter asiaticus genome [61]. In addition to computational prediction analysis, we obtained annotation data from a transcriptome profile study that contrasted tolerant and susceptible plants; *Citrus clementina* genes that were differently expressed via treatments and associated with illness were mined [62]. We were able to retrieve more than a dozen articles over the previous five years because of this manual literature mining, demonstrating the relevance of HLB research.

### 4.6. GreeningDB Implementation and Architecture

The database *GreeningDB* is hosted at USU’s Bioinformatics Facility using a Linux virtual machine within a high-performance computational cluster. The backend of the database was written in PHP, an open-source server-side scripting language, and implemented through an Apache server. Front-end visualization was written using HTML5, Bootstrap 4, and JavaScript.

**Figure 3:**
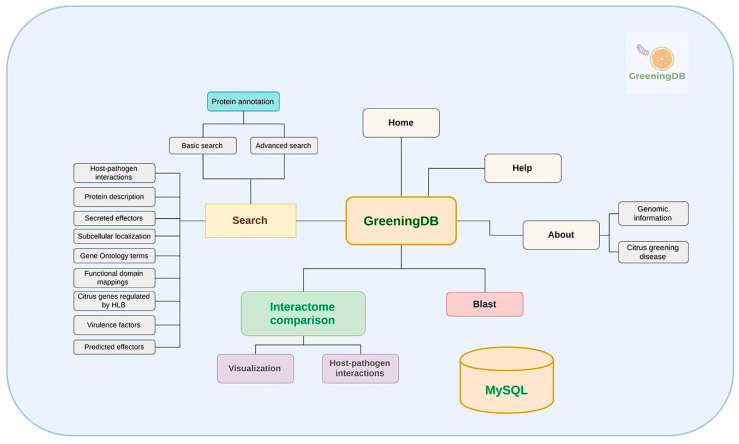
An overall view of the *GreeningDB* database architecture.

For the backend, all the PPI databases listed in section “*Protein–Protein interaction database collection*” were downloaded and installed locally as separate MySQL tables. Similarly, each of the features described in the section “*Features in GreeningDB*” were implemented locally as independent MySQL tables. The interolog prediction backend was implemented by combining in-house PHP and R scripts with SQL databases. PPI networks were visualized using SigmaJS library dedicated to graph drawing (http://sigmajs.org, accessed on 20 December 2019). BLAST results were visualized using the BlasterJS plugin. The overall architecture of the database is depicted in Figure 3.

## 5. Conclusions

*GreeningDB* is a comprehensive database that offers the scientific community functional annotation and a complete collection of HPI characteristics for the majority of the sequenced *Liberibacter* proteomes. We have developed a unique host–pathogen “comparactomics” tool as part of this resource, which is a prediction platform that allows us to compare two HLB interactomes at the same time. We anticipate that *GreeningDB* will be a valuable resource for the citrus breeding and experimental biology communities, bolstering and accelerating molecular research aimed at mitigating the huanglongbing disease.

## Figures and Tables

**Table 1:** List of the *Liberibacter* proteomes that are integrated into GreeningDB. Five species within *Liberibacter* genus are included: *Ca*. Liberibacter asiaticus, *Ca*. Liberibacter africanus, *Ca*. Liberibacter americanus, *Ca*. Liberibacter solanacearum, and *L. crescens*. Sources of unpublished proteomes are provided in the form of NCBI BioProject identifiers.

Organism	Strain	Protein Sequences	Genome Size (M)	GC %	HLB Pathogen	Reference
*Candidatus* Liberibacter asiaticus	gxpsy	1075	1.27	36.6	Yes	[20]
	A4	1027	1.23	36.4	Yes	[21]
	psy62	1021	1.22	36.5	Yes	[22]
	JXGC	1040	1.22	36.4	Yes	BioProject: 376787
	Ishi-1	997	1.19	36.3	Yes	[23]
	AHCA1	1010	1.23	36.6	Yes	BioProject: 470611
	FL17	1029	1.28	36.5	Yes	BioProject: 269509
	YNJS7C	1073	1.26	36.6	Yes	BioProject: 488522
	YCPsy	1042	1.23	36.5	Yes	[24]
	SGCA5	1005	1.20	36.4	Yes	[25]
	TX2351	1026	1.25	36.5	Yes	BioProject: 361117
	HHCA	827	1.15	36.5	Yes	[26]
	SGCA1	499	0.23	36.3	Yes	BioProject: 470611
*Candidatus* Liberibacter africanus	PTSAPSY	1017	1.19	31.1	Yes	[27]
*Candidatus* Liberibacter americanus	Sao Paulo	945	1.18	31.1	Yes	[28]
	PWSP	925	1.20	31.1	Yes	[29]
*Candidatus* Liberibacter solanacearum	Clso-ZC1	1151	1.26	35.2	No	[30]
	LsoNZ1	1199	1.31	35.3	No	[31]
	FIN114	1124	1.25	35.2	No	[32]
	HenneA	1104	1.21	34.9	No	[31]
	RSTM	1182	1.29	35.1	No	[33]
	FIN111	1087	1.20	34.9	No	[32]
	ISR100	1210	1.30	35.0	No	BioProject: 427973
	R1	1112	1.20	35.3	No	[34]
*Liberibacter crescens*	BT-1	1235	1.50	35.4	No	[35]

## Data Availability

The data can be accessed at http://bioinfo.usu.edu/GreeningDB/ (accessed on 11 August 2021).
